# Internipple Distance and Internipple Index in Prepubertal Turkish Girls

**DOI:** 10.4274/jcrpe.galenos.2020.2019.0161

**Published:** 2020-09-02

**Authors:** Seda Erişen Karaca, Şengül Cangür, İlknur Arslanoğlu

**Affiliations:** 1 Düzce University Faculty of Medicine, Department of Pediatric, Düzce, Turkey; 2 Düzce University Faculty of Medicine, Department of Biostatisitics, Düzce, Turkey; 3 Düzce University Faculty of Medicine, Department of Pediatric, Division of Pediatric Endocrinology, Düzce, Turkey

**Keywords:** Internipple distance, internipple index, Turkey

## Abstract

**Objective:**

To determine internipple distance and internipple index in prepubertal Turkish girls.

**Methods:**

The internipple distance and chest circumference of 667 healthy prepubertal Turkish girls aged 6 to 11 years were measured in a school screening program in Düzce. Measurements were performed at the end of expiration with a standard non-stretch tape measure graduated in millimeters with the arms hanging in a relaxed position on the sides of the body. The internipple distance was measured between the centers of both nipples, and chest circumference was measured across the internipple line. The internipple index was calculated by dividing the internipple distance (cm) x100 by the chest circumference (cm). Age specific internipple index reference curves were constructed and smoothed with the Lambda-Mu-Sigma method. Mean and standard deviations of internipple distance and internipple index were calculated according to decimal ages.

**Results:**

Age was found to be positively correlated with internipple distance and chest circumference, while it was negatively correlated with internipple index. The reference values of internipple index, including 3^rd^, 10^th^, 25^th^, 50^th^, 75^th^, 90^th^, and 97^th^ percentiles, and standard deviations were calculated for prepubertal girls.

**Conclusion:**

The reference ranges provided by this study might be helpful for the evaluation of syndromic cases by serving as normative data for internipple index in prepubertal girls aged 6-11 years in Turkey although ethnic differences may affect applicability to other countries.

**What is already known on this topic?**

Increased internipple distance is a clinical feature in some dysmorphic syndromes which may be subjectively evaluated by the physician during examination. Some studies have produced normative data for objective comparison of this parameter with “normal” after measurement and usually proportioning to chest circumference. However, these studies are scarce, and even fewer evaluate subjects beyond the neonatal period.

**What this study adds?**

There are no reference values available for a Turkish population. This study provides the first references for internipple distance and the internipple index in the country. In addition this reference data may serve internationally for school aged girls. A short overview of the literature is also given to illuminate the present status for these parameters.

## Introduction

Anthropometric evaluation of nipple placement may be helpful in diagnosing some syndromes. For instance, increased internipple distance is seen especially in Turner syndrome (TS), Noonan syndrome, fetal hydantoin syndrome, deletion 9p syndrome, Trisomy 8, Trisomy 18 and bilateral renal hypoplasia, while it may also be found in cerebro-oculo-facio-skeletal syndrome, Fraser syndrome, Bartsocas-Papas syndrome, Juberg-Hayward syndrome and Langer-Giedion syndrome (1). On the other hand, decreased internipple distance is generally reported in Jeune syndrome (asphyxic thoracic dystrophy) and cerebro-costo-mandibular syndrome (2).

TS is rather common with a frequency of one case per 2.500 live female births. However, diagnosis may be delayed due to the subtlety of dysmorphic features; in fact, it is not unusual for girls with TS to only be recognized in the prepubertal or pubertal period (3,4). TS should be considered in girls with short stature or primary amenorrhea. However, it is often overlooked in girls with short stature as shown research that found that up to 4% of girls who underwent genetic analysis due to short stature were found to have TS (5,6). Early diagnosis and prompt treatment are crucial in TS to prevent delayed intervention, growth disturbance, cognitive limitations, osteoporosis and to identify severe cardiac malformations (mostly aorta coarctation and bicuspid aortic valve) (7,8,9).

It was claimed that widened internipple distance is “illusionary” in TS, but the study supporting this idea was conducted in 36 syndromic and 247 normal children of both sexes which are small samples (10). Moreover, though the contribution to the diagnosis is not well known, subjective evaluation of internipple distance is almost always recorded in the clinical records of such cases. Thus an objective comparison of this stigma may increase the quality of the clinical examination in several syndromes. The evaluation of TS is part of the daily practice of pediatric endocrinologists. In addition to the contribution reference data for internipple distance may make to diagnosis, it is important as well for research purposes. Therefore to attempt to accurately measure internipple distance in girls and compare with normative data might prove to be a useful tool. However, data regarding internipple index and internipple distance are very limited in the literature. Considering that racial differences significantly affect internipple distance and internipple index, our aim with this study was to determine reference values for internipple distance and internipple index in prepubertal Turkish girls.

## Methods

The study was conducted between March-May 2009 in the context of a large school survey on 9.177 children in the 1^st^ to 8^th^ grade from 14 schools. The aim of this survey was to determine local height, weight and pubertal development data and finally to extract cases with short stature and puberty precox who were then invited to the hospital for a complete endocrine work-up (11). The schools were selected by stratified sampling method among 29 schools located in the city center of Düzce, Turkey, a small city located in North-West Anatolia, in pleasant wooded countryside. The stratification was made according to the socioeconomic level of the districts and the schools with highest student numbers were selected. A sample of 667 girls aged between 6-11 years were randomly selected from the schools and distributed as evenly as possible by age and group size in the present study. In every school the students came in order starting from the first to the sixth level. Each level was divided in four subgroups (classes). Approximately 50 girls in each school and nine from each level were targeted for screening. The first class to be sampled fulfilled the target number on most occasions. Birth date, date of physical examination, measured height, weight, internipple distance, chest circumference and pubertal scores according to Marshall and Tanner (12) were recorded and decimal age was calculated. Internipple distance and chest circumference were measured with a non-flexible tape at the end of expiration and with the arms hanging loosely by the sides. An experienced pediatric endocrinologist evaluated puberty and measured internipple distance and chest circumference. Height and weight were measured by a pediatrician. All measurements were made with light clothing, barefoot; internipple-chest measurements were made with naked upper body in a separate room. Internipple index was calculated using the following formula: Internipple distance (cm) x 100/chest circumference. Subjects with body mass index (BMI) above the 95th percentile, children with congenital anomalies or with a chronic disease were excluded from the study. Girls with thelarche were noted and excluded from the study (Tanner stage ≥B2).

The study protocol was approved by the Ethics Committee of Düzce University (protocol number: 2008.211/2189) and permission was obtained. Parents’ written consent was obtained prior to the study and the procedures were in accordance with those outlined by the Declaration of Helsinki.

### Statistical Analysis

The Statistical Package for the Social Sciences, version 22 (IBM Inc., Armonk, NY, USA) and the Lambda-Mu-Sigma (LMS) Chart Maker Pro version 2.54 software (13) were used for analyses. Descriptive statistics of the variables in the study were calculated. The normality assumption of continuous quantitative variables was checked with the Kolmogorov-Smirnov test. Mean, standard deviation, and minimum and maximum values were given as descriptive statistics of the variables in text and tables, since all quantitative variables met the normal distribution assumption. The relationships between quantitative variables were analyzed with the Pearson correlation test. The age-specific internipple index reference curves were constructed and smoothed with the LMS method in which the final curves of percentiles were produced by three smooth curves represented as L (Lambda, skewness), M (Mu, median) and S (Sigma, coefficient of variation) (14). Reference values of internipple index, including 3^rd^, 10^th^, 25^th^, 50^th^, 75^th^, 90^th^, and 97^th^ percentiles were determined. Statistical significance was accepted when p values were <0.05.

## Results

The mean age of the 667 individuals included in the study was 8.2±1.2 (6.3-11.5) years. Descriptive statistics for weight, height, BMI, internipple distance, chest circumference and internipple index for each decimal age are given in Table 1 and Table 2. As the subjects ages increase the sample number in groups decreased as the proportion of pubertal cases increased.

**Figure 1:**
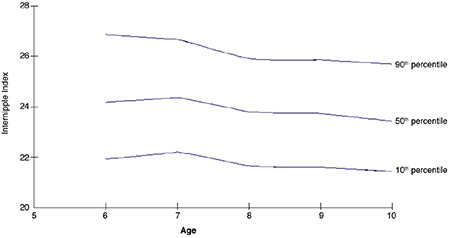
Graph showing the internipple index values (10^th^, 50^th^ and 90^th^ percentile), calculated using the Lambda-Mu-Sigma method, according to age

The internipple index values were estimated with the LMS method according to age and are presented in Table 3 along with L (lambda-skewness), M (median) and S (coefficient of variation) values for each age group. The graphical presentation of results is shown in Figure 1. These latter analyses were made among 657 subjects after removing the 11 year-old age group.

Age was found to be positively correlated with weight (r=0.491; p<0.001), height (r=0.660; p<0.001), internipple distance (r=0.158; p<0.001) and chest circumference (r=0.412; p=0.001); whereas it was negatively correlated with internipple index (r=-0.176; p<0.001). There was no relationship between age and BMI values (r=0.041; p=0.295). However, BMI values were found to be positively correlated with internipple distance (r=0.404; p<0.001) and chest circumference (r=0.628; p<0.001), while they were negatively correlated with internipple index (r=-0.084; p=0.030). Internipple index values were also negatively correlated with weight (r=-0.187; p<0.001) and height (r=-0.190; p<0.001).

## Discussion

The majority of studies assessing internipple index are comprised of patients in the newborn period (Table 4) (15,16,17,18,19,20,21). The newborn period is an important time and opportunity for recognizing dysmorphic syndromes. Therefore, these studies are valuable references but performing end-expiratory measurements in newborns and infants is almost impossible, while it is difficult in small children. Measurements performed with these limitations may cause erroneously high chest circumference and thus decreasing internipple index values. Therefore, studies with larger subject numbers are more reliable as measurement errors are balanced by the number of subjects.

Unfortunately, there are very few studies in older age groups (10,19,22). All identifiable studies involving older age groups have been summarized below. Chest measurement is easier in school aged girls since they cooperate quite well with instructions. However when puberty starts, the contribution of breast tissue to this parameter changes individually. Therefore, measurement is the easier to standardize and less prone to error in prepubertal girls. Tanner stage 1 girls were chosen for the present study resulting in the age distribution of our study. This age group is well representative of the age interval in which a great majority of TS patients attend clinics.

Feingold and Bossert (22) screened 2.403 children (2006 Caucasian, 206 Black and 43 Asian) between the newborn period and 14 years of age for many anthropometric indices. They reported that internipple index was the highest in the newborn period, with the lowest values between 15 months and seven years of age. This finding was partly supported by a study by Chen et al (1) who also found that internipple index was maximum at birth and decreased until two years of age.  Furthermore, in a study by Leung et al (19) internipple index decreased steadily from 1 to 18 years of age. Pelz (20) claimed that the intermammillary index, which is identical to internipple index, does not change with age prior to puberty.

In our study, this gradual decrease in internipple index was evident. Notably, the difference between the 3^rd^ and 97^th^ percentiles was the biggest in the age 6 group, it decreased somewhat in the age 7 group, but stabilized in the 8-10 groups. These groups show minor differences in percentiles between 5 and 95, which is perhaps ignorable. Correlation analyses was used to investigate the internipple index-age relationship and there was a negative correlation between these two parameters. There was also a negative correlation between both weight and height with internipple index. BMI was also negatively correlated with internipple index but this relationship was much weaker. Weight and height generally increase with age in childen although BMI is derived from these two parameters, so would be expected to remain relatively constant throughout the ages groups studied, given proportional increases in height and weight. In the study of Leung et al (19) the decrease in internipple index with age was relatively stable between the ages of 6 to 11 years. When we compared our 6-11 age results with the corresponding results of Leung et al (19), we found that internipple index values in in our study group were increasing with decreasing age by more than 1%, the variation in the same age groups was around 0.1% in the study of Leung et al (19). We can speculate that they couldn’t catch this age pattern with their limited subject numbers in each age group.

There are different views on the influence of ethnicity on internipple index measurements (1,16,19,20,21,22). In addition to ethnicity, methodologies and measurement devices may also contribute to these differences in internipple distance. On review of the literature, it was evident that Chinese neonates had higher internipple indexes than those reported in US, Nigerian and Hungarian cohorts (1,17,18).

The contribution of increased internipple distance to the diagnoses of Turner and Noonan patients was questioned in some earlier studies (1,10). Chen et al (1) found that internipple distance in patients with TS (n=40) was significantly different (p=0.001) from normal after adjusting for height but not for age. However, the difference between TS and unaffected subjects was less striking when compared to chest width or circumference (p=0.01), but still significant Collins (10) suggested that an increased internipple distance is a subjective clinical impression owing to the illusion created by the body shape and short stature in Turner as well as Noonan syndrome of note, samples sizes in these two studies were quite small and their hypotheses should be tested in larger groups of Turner and Noonan patients in comparison to normal subjects. Since height is effective on both the objectively measured internipple index as shown in our study and on perceived internipple distance as claimed in Collin’s study, it might be reasonable to compare Turner subject’s height age with normative data, rather than chronological age.

### Study Limitations

Sample numbers in older age groups were reduced due to the increasing proportion of pubertal cases.

## Conclusion

Although the internipple index provides a method of assessment that is objective in comparison to physical examination alone, and could increase the chances of early diagnosis in many syndromes, especially in TS, it is apparent that racial and ethnic differences may cause variations in assessment. Therefore, appropriate reference intervals should be used. In this study, normal values of internipple distance and internipple index were obtained for the Turkish population in prepubertal girls aged between 6-11 years. Despite the possibility of contribution of ethnic differences to a variation in internipple index, our data might be used as reference values for other countries with largely Caucasian populations, until local normative values are available. Regarding our local population, the missing neonatal reference standards might be helpful additional data when future studies of internipple distance are performed.

## Figures and Tables

**Table 1:**
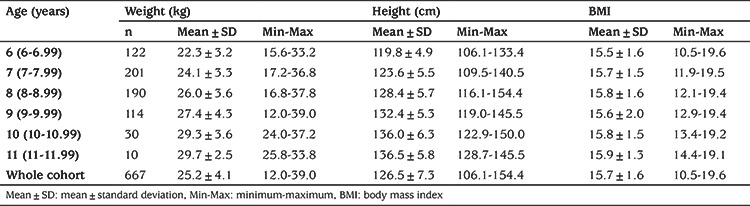
Mean anthropometric values of age groups

**Table 2:**
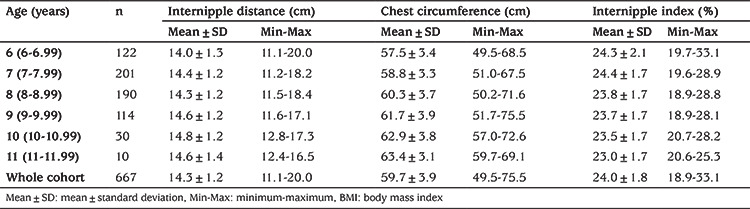
Internipple distance and internipple indices with regard to age groups

**Table 3:**
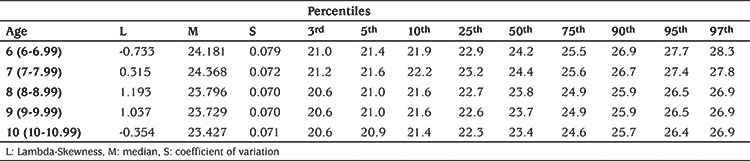
Smoothed age-specific internipple index percentile values of prepubertal girls aged 6 to 10 years

**Table 4:**
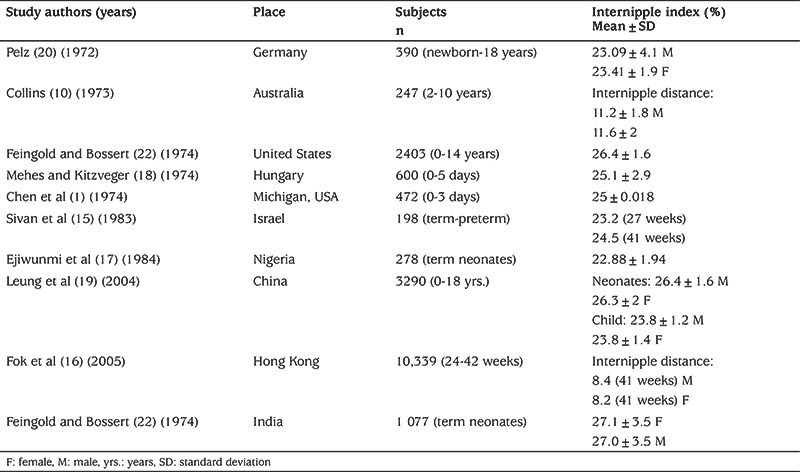
Internipple distances and internipple indices in other studies

## References

[1] Chen H, Espiritu C, Casquejo C, Boriboon K, Whoolley P Jr (1974). Inter-nipple distance in normal children from birth to 14 years and in children with Turner’s, Noonan’s, Down’s and other aneuploides. Growth.

[2] Merlob P (2003). Congenital malformations and developmental changes of the breast: a neonatologist view. J Pediatr Endocrinol Metab.

[3] Gürsoy S, Erçal D (2017). Turner Syndrome and Its Variants. J Pediatr Res.

[4] Morgan T (2007). Turner Syndrome: Diagnosis and Management. Am Fam Physician.

[5] Savendahl L, Davenport ML (2000). Delayed diagnoses of Turner syndrome: Proposed guidelines for change. J Pediatr.

[6] Moreno-Garcia M, Fernandez-Martinez FJ, Barreiro Miranda E (2005). Chromosomal anomalies in patients with short stature. Pediatr Int.

[7] Völkl TMK, Degenhardt K, Koch A, Simm D, Dörr HG, Singer H (2005). Cardiovascular anomalies in children and young adults with Ullrich-Turner syndrome the Erlangen experience. Clin Cardiol.

[8] Elsheikh M, Casadei B, Conway GS, Wass JA (2001). Hypertension is a major risk factor for aortic root dilatation in women with Turner syndrome. Clin Endocrinol (Oxf).

[9] Mazzanti L, Cacciari E (1998). Congenital heart disease in patients with Turner’s syndrome. Italian Study Group for Turner Syndrome (ISGTS). J Pediatr.

[10] Collins E (1973). The illusion of widely spaced nipples in the Noonan and the Turner syndromes. J Pediatr.

[11] Arslanoğlu I, Topçu B, Özkul-Tandoğan M, Coşgun E, Oğuzhan T, Turan S, Yavuz T, Uzun H, Şenses DA, Kocabay K (2009.). Düzce İl Merkezi İlköğretim Öğrencisi Kız Çocuklarında Meme Evreleri ve Yaş İlişkisi. XIII. Ulusal Pediatrik Endokrinoloji Kongresi Kitabı, 137, Antalya.

[12] Marshall WA, Tanner JM (1969). Variations in pattern of pubertal changes in girls. Arch Dis Child.

[13] Pan H, Cole TJ. LMS Chartmaker. 2011. Last accessed date: Agust, 2019). Available at:.

[14] Cole TJ, Green PJ (1992). Smoothing reference centile curves: The LMS method and penalized likelihood. Stat Med.

[15] Sivan Y, Merlob P, Reisner SH (1983). Sternum length, torso length, and internipple distance in newborn infants. Pediatrics.

[16] Fok TF, Hon KL, Wong E, Ng PC, So HK, Lau J, Chow CB, Lee WH;, Hong Kong Neonatal Measurements Working Group (2005). Trunk anthropometry of Hong Kong Chinese infants. Early Hum Dev.

[17] Ejiwunmi AB, Okanlawon OA, Ojo OO (1984). Interpupillary and internipple distances and ear lengths in Nigeran newborns. Ann Trop Paediatr.

[18] Mehes K, Kitzveger E (1974). Inner canthal and intermamillary indices in the Newborn infant. J Pediatr.

[19] Leung AK, Kao CP, Sauve RS, Fang JH, Leong AG, Liu EK (2004). Internipple distance and internipple index. J Natl Med Assoc.

[20] Pelz L (1972). Intermammillary index in children. Kinderarztl Prax.

[21] Faridi MMA, Dhingra P (2013). Internipple measurements in Indian neonates. S Afr J CH.

[22] Feingold M, Bossert WH (1974). Normal values for selected physical parameters: an aid to syndrome delineation. Birth Defects Orig Artic Ser.

